# Study of Stroke Incidence in the Aseer Region, Southwestern Saudi Arabia

**DOI:** 10.3390/ijerph15020215

**Published:** 2018-01-26

**Authors:** Adel A. Alhazzani, Ahmed A. Mahfouz, Ahmed Y. Abolyazid, Nabil J. Awadalla, Razia Aftab, Aesha Faraheen, Shamsun Nahar Khalil

**Affiliations:** 1 Department of Internal Medicine, College of Medicine, King Khalid University, P.O. Box 641, Abha 61421, Saudi Arabia; alhazzani@kku.edu.sa; 2 Department of Family and Community Medicine, College of Medicine, King Khalid University, P.O. Box 641, Abha 61421, Saudi Arabia; drzizous2000@yahoo.com (A.Y.A.); njgirgis@yahoo.co.uk (N.J.A.); drraziaaftab@gmail.com (R.A.); draeshasiddiqui@gmail.com (A.F.); shamsun203@gmail.com (S.N.K.); 3 Department of Epidemiology, High Institute of Public Health, Alexandria University, Alexandria 21511, Egypt; 4 Department of Community Medicine, College of Medicine, Mansoura University, Mansoura 35516, Egypt

**Keywords:** stroke, incidence, Saudi Arabia

## Abstract

Recent data regarding first-stroke incidence in Saudi Arabia in general and in the Aseer region in particular are scarce and even lacking. The aim of this work was to study the first-time stroke incidence in the Aseer region, southwestern Saudi Arabia. All first-stroke patients admitted to all hospitals in the Aseer region over a one-year period (January through December 2016) were included. Stroke patients outside the Aseer region were excluded from the study. The incidence per 100,000 patients and the concomitant 95% CI (Confidence Intervals) were computed. The present study included 1249 first-time stroke patients and calculated an overall minimal incidence rate of hospitalized first-time stroke of 57.64 per 100,000 persons per year (95% CI: 57.57–57.70). A steady increase was noticed depending on the patients' age, reaching a figure of 851.81 (95% CI: 849.2–854.5) for those patients aged 70 years and more. Overall, the incidence rate for females (48.14; 95% CI: 48.04–48.24) was lower compared to males (65.52; 95% CI: 65.1–66.0). Taking into consideration the expected rise of the elderly because of the prominent medical services provided by the Saudi government, leading to a subsequent change in the horizontal and vertical age distribution structure of the population, an increase in the number of stroke patients is expected. It is suggested to establish a nationwide stroke surveillance system in the Kingdom, with the objective to report, analyze, and maintain an updated overview of the stroke status in Saudi Arabia.

## 1. Introduction

Globally, the burden of stroke has increased rapidly over the past two decades [1]. Stroke has been estimated as the second leading cause of death and disability-adjusted life years [2,3]. Current epidemiological data indicate that 16.9 million people suffer a stroke each year, providing a global incidence of 258/100,000 persons/year and accounting for 11.8% of total deaths worldwide [3,4].

The Middle East region faces a variable burden of stroke. The incidence rate for all strokes ranged between 22.7 and 250 per 100,000 people per year in this region [5]. Because of the dramatic transformation of the social, economic, and environmental conditions over the past few decades in this region, the lifestyle has changed rapidly, which has caused a transition to a high burden of stroke. 

Studies in Saudi Arabia have provided a hospital-based crude annual incidence rate of stroke of 15.1 per 100,000 persons in Jizan [6], 29.8 per 100,000 persons in the Eastern province [7], and 43.8 per 100,000 persons in Riyadh [8].

The Aseer region is located in the southwest of Saudi Arabia, covering an area of more than 80,000 km^2^. The region extends from the high mountains of Sarawat (with an altitude of 3200 m above sea level) to the Red Sea and lies few kilometers from the northern border of neighboring Yemen. The region is bordering Jizan and is located to its northeast.

A national stroke registry is not available in Saudi Arabia. Recent data regarding the first-stroke incidence in Saudi Arabia in general and in the Aseer region in particular are scarce and even lacking. The aim of the present study was to study the first-time stroke incidence in the Aseer region, southwestern Saudi Arabia. 

## 2. Materials and Methods

The last reported Aseer region population is 2,166,983 [9]. Health services delivery in the region is provided by a network of 242 primary health-care centers, 12 secondary-care hospitals, and one tertiary hospital (Aseer central hospital).

All newly admitted first-stroke cases in the study hospitals were diagnosed, and their records were revised by neurologists. The stroke case definition adopted in the present study described stroke as a focal neurological deficit due to cerebral infarction or hemorrhage, confirmed by computed tomography (CT) scan or Magnetic Resonance Imaging (MRI). This definition was based on Saudi Ministry of Health Guidelines (MOH Pocket Manual in Critical Care) which are originally based on International Statistical Classification of Diseases and Related Health Problems 10th Revision (ICD-10). All first-stroke patients admitted to all hospitals in the Asser region over a one-year period (January through December 2016) were included. Stroke patients outside the Aseer region were excluded from the study. The data were collected by hospital neurologists and trained research nurses.

The study was conducted in accordance with the Declaration of Helsinki, and the protocol was approved by the Ethics and Research Committee of the College of Medicine, King Khalid University.

The data were analyzed by using SPSS, version 22. Proportions (incidence per 100,000) and concomitant 95% confidence intervals (95% CI) were computed. When comparing the 95% CI in males and females, statistically significant differences were considered if the two confidence intervals did not overlap. The Pearson chi square (χ^2^) test was used as a test of significance to compare different age groups; *p*-values ≤ 0.05 was considered as statistically significant.

## 3. Results

The present study included 1249 first-time stroke patients admitted to the study hospitals during the study period from 1 January 2016 till 31 December 2016. The cases were from all hospitals in Aseer. Taking into consideration the last reported Aseer region population of 2,166,983 [9], this figure gave us an overall minimal incidence rate of hospitalized first-time stroke of 57.64 per 100,000 person per year (95% CI: 57.57–57.70 per 100,000). 

**Figure 1:**
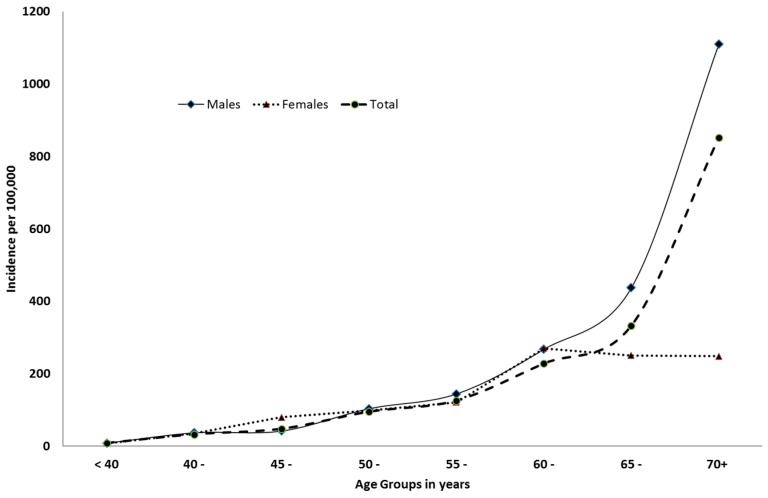
Minimal incidence rate of hospitalized first-time stroke (per 100,000 people) by sex and age groups.

Table 1 and Figure 1 show the minimal incidence rate of hospitalized first-time stroke patients by sex and age groups. The minimal incidence rate for patients aged less than 40 years was 8.41 per 100,000 people (95% CI: 8.37–8.46). The figure increased to reach 32.66 per 100,000 (95% CI: 32.44–32.88) for patients aged 40 to 44 years. Similarly, the figure increased to reach 47.83 per 100,000 (95% CI: 47.56–48.09) for patients aged 45–49 years. A steady increase was noticed depending on the age, reaching a figure of 851.81 per 100,000 (95% CI: 849.2–854.5) for patients aged 70 years and more (with a mean of 80.3 ± 8.2 years and a median of 80 years). The steady increase was statistically significant (*p* = 0.001).

**Figure 2:**
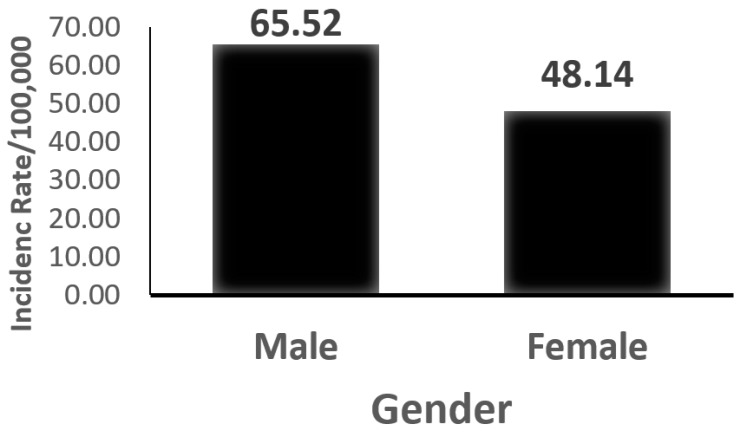
Minimal incidence rate of hospitalized first-time stroke (per 100,000 people) by gender.

Regarding the incidence rate by gender (Figure 2 and Table 1), the incidence rate for females was 48.14 per 100,000 (95% CI: 48.04–48.24). This figure was lower if compared to males (65.52 per 100,000; 95% CI: 65.1–66.0). The difference was statistically significant (Pearson Chi square = 28.14, *p* = 0.001). A similar trend of significant lower incidence rates among females compared to males was observed for most of the studied age groups (less than 40, 40–44, 50–54, 55–59, 60–64, 65–69, and 70+). On the other hand, in the age group 45–49, the incidence rate for males was significantly lower than that for females. No significant difference was found in the age group 60–64. The increase in incidence by age was observed in both males and females. In females, the incidence was almost stationary over the age of 60 (Figure 1).

## 4. Discussion

Stroke is a significant public health issue worldwide [2]. A recent publication regarding the systematic analysis for the Global burden of disease 2015 showed that neurological disorders accounted for 10.2% of global DALYs (disability-adjusted life years) and stroke accounted for 47.3% of the overall neurological disorders burden. Stroke ranked first among age-standardized DALY rates for neurological disorders globally and in the Middle East countries [10].

The incidence of stroke is increasing in Asia, particularly in the Middle Eastern region. This region faces a high burden of stroke because of the growing rates of non communicable diseases [5]. In the Kingdom of Saudi Arabia, stroke is a rapidly growing problem and a major cause of illness and death. This increasing incidence is due to the changing life style in the country and high prevalence of diabetes mellitus, obesity, dyslipidemia, and hypertension, all considered to be important risk factors for stroke [5].

The present study revealed an overall incidence rate of first-time stroke of 57.64 per 100,000 person per year (95% CI: 57.3–57.9 per 100,000) in the Aseer region. A literature review in Middle East countries showed a lack of recent studies [5]. First-time stroke incidence studies in Kuwait showed a figure of 27.59 per 100,000 persons in 1997 [11]. A figure of 41 per 100,000 persons was reported in Qatar in 2001 [12]. In Bahrain, in 2000, a figure of 57 per 100,000 persons was reported [13]. In Iran, a systematic review revealed an annual stroke incidence ranging from 23 to 103 per 100,000 persons [14]. In Libya, in 1995, a figure of 48 per 100,000 persons was reported [15]. These figures show that in Middle East countries the incidence varied from 27.5 per 100,000 persons per year in Kuwait [11] to 103 per 100,000 persons in Iran [14]. In interpreting these figures, the different methodologies adopted in these studies should be taken in consideration [5].

In Saudi Arabia, a literature review revealed a similar lack of recent publications regarding the incidence of first-time stroke studies. In 1993, a figure of 43.8 per 100,000 people was reported [8]. In 1998, a figure of 29.8 per 100,000 people was reported [16]. It is worth mentioning that the first article was a retrospective register study and the second one was a prospective study. A recent publication conducted in Al-Madinah Al-Munawarah city, Saudi Arabia, in 2014 reported an incidence rate of first-time stroke of 13.89 per 100,000 persons [17]. This figure in Al-Madinah is much lower than the figure of the present study in Aseer (57.64). The difference could be explained by the fact that the Aseer study included almost all hospitals in the region, while the Al-Madinah study included only one hospital.

Similar low figures of the incidence of stroke per 100,000 persons were reported in less developed countries, including Malaysia (67) [18], Brazil (86.9) [19], and India (145) [20]. On the other hand, high figures were reported in developed countries like the Czech Republic (241) [21], Japan (290.3) [22], and Sweden (314) [23]. A recent article in 2016, reporting the Global Burden of Disease 2010 study, showed marked differences in stroke incidence between high-income (217/100,000/year) and low-income (281/100,000/year) countries [4].

The present study reported a significant difference in the incidence of stroke in the Aseer region depending on the gender. Generally, females had a significant lower incidence rate compared to males. Different studies in the USA reported similar trends, where males were more likely to suffer a stroke as compared to females [24,25]. The same trend was reported in Hong Kong [26], New Zealand [27], and in the global burden of disease study 2013 [28]. In our series, this trend of male preponderance was observed in almost all age groups except for the age group 45–49, for which the incidence was higher among females. The preponderance of males in our series goes hand in hand with the results of different studies [29]. The female preponderance in the age group 45–49 may be explained by the start of the decline in the protective sex hormones [29]. Yet, further studies are needed to probe this age group in the Aseer region.

## 5. Conclusions

In conclusion, the present study revealed an overall minimal incidence rate of hospitalized first-time stroke of 57.64 per 100,000 person per year in the Aseer region. This figure increased to reach 108.05 per 100,000 for those patients aged 50–59 years. A steady increase was noticed depending on the age, reaching a figure of 1211.41 per 100,000 for those patients aged 80 years and more. Taking into consideration the well-known fact that a large sector of the Saudi population is younger than 30 years, the eventual expected rise of the elderly as a result of the distinguished medical services provided by the Saudi government, and the increased rate of breeding, there is an expected change in the horizontal and vertical age distribution structure of the population, with an accompanying increase of the elderly population and, thus, an increase in the number of stroke victims. Based on the results of this study, it is suggested to establish a nationwide stroke surveillance system in the Kingdom, with the objective is to report, analyze, and maintain an updated overview of the stroke status in Saudi Arabia.

## Figures and Tables

**Table 1:** Minimal incidence rate of hospitalized first-time stroke patients (per 100,000 people) and concomitant 95% Confidence Intervals (95% CI) by gender and age groups.

Age Group	Males		Females		Total	
	Cases/Pop.	Incidence per 100,000 (95% CI)	Cases/Pop.	Incidence per 100,000 (95% CI)	Cases/Pop.	Incidence per 100,000 (95% CI)
<40	70/813,170	8.61 (8.55–8.67) *	59/719,814	8.20 (8.13–8.26) *	129/1,532,984	8.41 (8.37–8.46)
40–44	40/107,428	37.23 (36.95–37.48) *	18/50,481	35.66 (35.24–36.08) *	58/177,576	32.66 (32.44–32.88)
45–49	36/85,422	42.14 (41.81–42.48) *	29/36,169	80.18 (79.77–80.59) *	65/135,903	47.83 (47.56–48.09)
50–54	62/59,683	103.88 (101.4–106.4) *	29/29,236	99.19 (99.08–99.29) *	91/95,852	94.94 (94.80–95.08)
55–59	60/41,509	144.55 (141.2–148.0) *	29/23,904	121.32 (117.2–125.5) *	89/70,745	125.80 (123.04–128.30)
60–64	77/28,742	267.90 (262.8–273.1)	43/15,902	270.41 (263.6–277.4)	120/52,646	227.94 (216.0–241.0)
65–69	70/15,994	437.66 (430.0–445.4) *	36/14,382	250.31 (257.6–272.4) *	106/31,896	332.33 (313.0–353.0)
70+	361/32,512	1110.36 (1076.0–1145.0) *	230/92,635	248.28 (238.0–259.0) *	591/69,381	851.81 (849.2–854.5)
Total	776/1,184,460	65.52 (65.1–66.0) *	473/982,523	48.14 (48.04–48.24) *	1249/2,166,983	57.64 (57.57–57.70)

Pop. = Population in the Aseer region by age in mid-2016 (demographic survey 2016, The Saudi General Authority for Statistics). * Significant differences in the incidence by gender, based on 95% CI (if the two confidence intervals did not overlap).

## References

[1] Murray C.J., Vos T., Lozano R., Naghavi M., Flaxman A.D., Michaud C., Ezzati M., Shibuya K., Salomon J.A., Abdalla S. (2013). Disability-adjusted life years (dalys) for 291 diseases and injuries in 21 regions, 1990–2010: A systematic analysis for the global burden of disease study 2010. The Lancet.

[2] Thrift A.G., Thayabaranathan T., Howard G., Howard V.J., Rothwell P.M., Feigin V.L., Norrving B., Donnan G.A., Cadilhac D.A. (2017). Global stroke statistics. Int. J. Stroke.

[3] Benjamin E.J., Blaha M.J., Chiuve S.E., Cushman M., Das S.R., Deo R., de Ferranti S.D., Floyd J., Fornage M., Gillespie C. (2017). Heart disease and stroke statistics—2017 update: A report from the American heart association. Circulation.

[4] Béjot Y., Delpont B., Giroud M. (2016). Rising stroke incidence in young adults: More epidemiological evidence, more questions to be answered. Am. Heart Assoc..

[5] El-Hajj M., Salameh P., Rachidi S., Hosseini H. (2016). The epidemiology of stroke in the middle east. Eur. Stroke J..

[6] Ayoola A.E., Banzal S.S., Elamin A.K., Godour M., Elsammani E.W., Al-Hazmi M.H. (2003). Profile of stroke in Gazan, kingdom of Saudi Arabia. Neurosciences.

[7] Robert A.A., Zamzami M.M. (2014). Stroke in Saudi Arabia: A review of the recent literature. Pan Afr. Med. J..

[8] Al Rajeh S., Awada A., Niazi G., Larbi E. (1993). Stroke in a Saudi Arabian national guard community. Analysis of 500 consecutive cases from a population-based hospital. Stroke.

[9] Central Authority of Statistics, Kingdom of Saudi Arabia. Statistical Yearbook of 2016.

[10] Feigin V.L., Abajobir A., Abate K., Abd-Allah F., Abdulle A., Abera S., Abyu G., Ahmed M., Ärnlöv J., Vos T. (2017). Global, regional, and national burden of neurological disorders during 1990–2015: A systematic analysis for the global burden of disease study 2015. Lancet Neurol..

[11] Abdul-Ghaffar N.U., El-Sonbaty M.R., Abdul-Baky M.S.E.-D., Marafie A.A., Al-Said A.M. (1997). Stroke in kuwait: A three-year prospective study. Neuroepidemiology.

[12] Hamad A., Hamad A., Sokrab T.E.O., Momeni S., Mesraoua B., Lingren A. (2001). Stroke in Qatar: A one-year, hospital-based study. J. Stroke Cerebrovasc. Dis..

[13] Al-Jishi A., Mohan P.K. (2000). Profile of stroke in Bahrain. Neurosciences.

[14] Hosseini A.A., Sobhani-Rad D., Benamer H.T., Ghandehari K. (2010). Frequency and clinical patterns of stroke in iran-systematic and critical review. BMC Neurol..

[15] El Zunni S., Ahmed M., Prakash P.S., Hassan K.M. (1995). Stroke: Incidence and pattern in Benghazi, Libya. Ann. Saudi Med..

[16] Al-Rajeh S., Larbi E.B., Bademosi O., Awada A., Yousef A., Al-Freihi H., Miniawi H. (1998). Stroke register: Experience from the eastern province of Saudi Arabia. Cerebrovasc. Dis..

[17] Al-Shenqiti A.M., Ibrahim S.R., Khaled O.A., Ali A.R.H., Ahmed M.S. (2017). Incidence of first time stroke: A Saudi experience. Eur. Neurol..

[18] Neelamegam M., Looi I., Cheah W.K., Narayanan P., Hamid A.M.A., Ong L.M. (2013). Stroke incidence in the south west district of the Penang island, Malaysia: Pearls: Penang acute stroke research longitudinal study. Prev. Med..

[19] Cabral N., Gonçalves A., Longo A., Moro C., Costa G., Amaral C., Fonseca L., Eluf-Neto J. (2009). Incidence of stroke subtypes, prognosis and prevalence of risk factors in Joinville, brazil: A two-year, community-based study. J. Neurol. Neurosurg. Psychiatry.

[20] Dalal P., Malik S., Bhattacharjee M., Trivedi N., Vairale J., Bhat P., Deshmukh S., Khandelwal K., Mathur V. (2008). Population-based stroke survey in Mumbai, India: Incidence and 28-day case fatality. Neuroepidemiology.

[21] Sedova P., Brown R.D., Zvolsky M., Kadlecova P., Bryndziar T., Kubelka T., Weiss V., Volný O., Bednarik J., Mikulik R. (2017). Incidence of hospitalized stroke in the Czech Republic: The national registry of hospitalized patients. J. Stroke Cerebrovasc. Dis..

[22] Omama S., Yoshida Y., Ogasawara K., Ogawa A., Ishibashi Y., Ohsawa M., Tanno K., Onoda T., Itai K., Sakata K. (2013). Incidence rate of cerebrovascular diseases in northern japan determined from the iwate stroke registry with an inventory survey system. J. Stroke Cerebrovasc. Dis..

[23] Appelros P., Nydevik I., Seiger Å., Terént A. (2002). High incidence rates of stroke in Orebro, Sweden: Further support for regional incidence differences within Scandinavia. Cerebrovasc. Dis..

[24] Rosamond W., Flegal K., Furie K., Go A., Greenlund K., Haase N., Hailpern S.M., Ho M., Howard V., Kissela B. (2008). Heart disease and stroke statistics—2008 update. Circulation.

[25] Turtzo L.C., McCullough L.D. (2010). Sex-specific responses to stroke. Future Neurol..

[26] Wu S., Ho S., Chau P., Goggins W., Sham A., Woo J. (2012). Sex differences in stroke incidence and survival in Hong Kong, 2000–2007. Neuroepidemiology.

[27] Dyall L., Carter K., Bonita R., Anderson C., Feigin V., Kerse N., Brown P. (2006). Auckland Regional Community Stroke (ARCOS) Study Group. Incidence of stroke in women in Auckland, New Zealand. Ethnic trends over two decades: 1981–2003. N. Z. Med. J..

[28] Barker-Collo S., Bennett D.A., Krishnamurthi R.V., Parmar P., Feigin V.L., Naghavi M., Forouzanfar M.H., Johnson C.O., Nguyen G., Mensah G.A. (2015). Sex differences in stroke incidence, prevalence, mortality and disability-adjusted life years: Results from the global burden of disease study 2013. Neuroepidemiology.

[29] Wilson M.E. (2013). Stroke: Understanding the differences between males and females. Pflügers Arch. Eur. J. Physiol..

